# Partial nitritation of stored source-separated urine by granular activated sludge in a sequencing batch reactor

**DOI:** 10.1186/s13568-017-0354-9

**Published:** 2017-02-28

**Authors:** Liping Chen, Xiaoxiao Yang, Xiujun Tian, Song Yao, Jiuyi Li, Aimin Wang, Qian Yao, Dangcong Peng

**Affiliations:** 1 Department of Municipal and Environmental Engineering, Beijing Key Laboratory of Aqueous Typical Pollutants Control and Water Quality Safeguard, Beijing Jiaotong University, Beijing, 100044 China; 2 School of Environmental and Municipal Engineering, Xi’an University of Architecture and Technology, Xi’an, 710048 China

**Keywords:** Source-separated urine, Partial nitritation, Nitrogen removal, Granular sludge, Fluorescence in situ hybridization

## Abstract

The combination of partial nitritation (PN) and anaerobic ammonium oxidation (anammox) has been proposed as an ideal process for nitrogen removal from source-separated urine, while the high organic matters in urine cause instability of single-stage PN-anammox process. This study aims to remove the organic matters and partially nitrify the nitrogen in urine, producing an ammonium/nitrite solution suitable for anammox. The organic matters in stored urine were used as the electron donors to achieve 40% total nitrogen removal in nitritation-denitrification process in a sequencing batch reactor (SBR). Granular aggregates were observed and high mixed liquor suspended solids (9.5 g/L) were maintained in the SBR. Around 70–75% ammonium was oxidized to nitrite under the volumetric loading rates of 3.23 kg chemical oxygen demand (COD)/(m^3^ d) and 1.86 kg N/(m^3^ d), respectively. The SBR produced an ammonium/nitrite solution free of biodegradable organic matters, with a NO_2_^−^–N:NH_4_^+^–N of 1.24 ± 0.13. Fluorescence in situ hybridization images showed that *Nitrosomonas*-like ammonium-oxidizing bacteria, accounting for 7.2% of total bacteria, located in the outer layer (25 μm), while heterotrophs distributed homogeneously throughout the granular aggregates. High concentrations of free ammonia and nitrous acids in the reactor severely inhibited the growth of nitrite-oxidizing bacteria, resulting in their absence in the granular sludge. The microbial diversity analysis indicated *Proteobacteria* was the predominant phylum, in which *Pseudomonas* was the most abundant genus.

**Electronic supplementary material:**

The online version of this article (doi:10.1186/s13568-017-0354-9) contains supplementary material, which is available to authorized users.

## Introduction

Source separation and treatment of human urine have attracted extensive interest in urban water management since urine constitutes at least 50% of total phosphorus (P) load and 80% of total nitrogen (N) load in municipal wastewater, but represents less than 1% of total volume (Hanæus et al. 1997; Maurer et al. 2006). Urine contains high concentration of nitrogen, up to 8000 mg-N/L, mainly in the form of urea, which is spontaneously hydrolyzed into ammonia nitrogen and bicarbonate, concurrently elevating the pH values over 9 (Udert et al. 2003). Owing to the high content of nutrients in source-separated urine, decentralized treatment could provide an opportunity for efficient nutrient removal options.

The combination of partial nitritation (PN) and anaerobic ammonium oxidation (anammox) has recently been regarded as a promising and energy efficient approach to remove the nitrogen compounds in high strength wastewaters (Kartal et al. 2010). In the PN process, half NH_4_^+^ is oxidized into nitrite (NO_2_^−^) by autotrophic ammonium-oxidizing bacteria (AOB) under the aerobic environment. In the subsequent anammox process, the remaining NH_4_^+^ is oxidized by NO_2_^−^ to di-nitrogen gas (N_2_) under the anaerobic conditions. The PN-anammox process can be established in a single reactor (single-stage) or two separated reactors (two-stage). In comparison to the conventional nitrification and denitrification, the combined PN-anammox process could save 63% oxygen requirement, and eliminate the addition of external organic carbon (Strous et al. 1998). PN-anammox process has also been proposed for nitrogen removal from urine (Wilsenach et al. 2007; Burgmann et al. 2011).

From the viewpoint of microbial composition, well-tuned bacterial communities consisting of AOB and anammox bacteria (AMX) are required to achieve stable operation of PN-anammox. The amount of nitrite-oxidizing bacteria (NOB) should be low to prevent the oxidization of nitrite to nitrate. Since the growth rates of AOB and AMX are far less than heterotrophic bacteria (HET), precise control of the growth of HET, which compete with AOB for oxygen and with AMX for nitrite, is of crucial importance. As a result, the influence of organic loading rates, or the chemical oxygen demand to nitrogen ratio (COD/N) in PN-anammox process has been investigated by many researchers. Complete inhibition of AOB under high COD conditions has been well observed by previous studies (Gomez et al. 2000). Chamchoi et al. (2008) reported that AMX were outcompeted by HET in an anoxic system when the COD/N ratio was higher than 2.0 g-COD/g-N. Udert and colleagues reported that high COD/N ratios (up to 1.4–1.5 g-COD/g-N) led to instability in PN-anammox systems treating synthetic wastewater and diluted urine, and the abundance and activity of AMX were negatively affected by the elevated organic loading rates (Udert et al. 2008; Burgmann et al. 2011; Jenni et al. 2014). So far, one-stage PN-anammox systems have mainly been operated with influent COD/N ratios below 0.5 g-COD/g-N. The feasible solution for high COD wastewater seems to reduce the nitrogen elimination rate, for instance 0.27 kg N/(m^3^ d) in Jenni et al. (2014). Source-separated urine contains high amounts of organic compounds (COD can be as high as 10 g/L, Udert et al. 2006) which implies two-stage PN-anammox be preferred. To apply anammox process in urine nitrogen removal, the biodegradable organic matters in urine should be effectively removed in the first PN stage to eliminate the adverse impacts on AMX. At the same time, the ammonium in the wastewater is partially oxidized to nitrite, and an effluent suitable for the second-stage anammox process is produced. If the organic matter could be utilized for nitrite reduction prior to anammox process, the overall nitrogen loads would be reduced as well.

Consequently, the objectives of this study were to: (1) investigate the effect of organic matters in urine on nitrogen removal; (2) produce an effluent with a suitable composition for anammox process and develop a treatment approach for decentralized N removal from stored urine.

## Materials and methods

### Urine and the feed to the sequencing batch reactor

Undiluted urine samples were collected in a barrel from a male toilet in a teaching building in Beijing Jiaotong University. The urine was harvested daily and stored in sealed containers (20 L) at room temperature for more than 2 months to achieve a fully hydrolyzed urine with a stable ammonium concentration. Prior to organic removal and PN, the P in stored urine was recovered by precipitation with a brine from reverse osmosis (RO) process as a precipitant (Tian et al. 2016). As described in Additional file 1, urine and RO brine were mixed at a volumetric ratio of 1:1 in a precipitation reactor, the effluent from which was fed to a laboratory-scale sequencing batch reactor (SBR) for both organic matter removal and PN. The main water quality of precipitation reactor effluent is listed in Table 1. Since real urine samples were used in the study, the wastewater quality varied in a considerably wide range.

**Table 1:** The main water quality of the wastewater used in the study

Parameter	Value^a^	Unit
pH	9.1–9.4	
COD	2200–4800	mg/L
Total nitrogen	1800–3500	mg/L
NH_4_^+^–N	1720-3380	mg/L
TP	11.5–25.3	mg/L
Alkalinity	7170–9850	mg CaCO_3_/L
Total dissolved solids	12,700–24,380	mg/L

^a^The values of the wastewater from the precipitation reactor

### Reactor and operation

The SBR made of a plexiglass cylinder (15 cm inner diameter) has an effective volume of 3.6 L. The reactor was started with suspended biomass from an activated sludge process treating municipal wastewater (Gaobeidian WWTP, Beijing, China). The initial concentration of volatile suspended solids (VSS) in the SBR was ~4.0 g/L. Each cycle of the SBR operation is 12 h, consisting of a feeding phase with stirring (30 min), an anoxic phase (3 h and 30 min), an aerobic phase (7 h), a settling phase (30 min), and effluent withdrawal (30 min). The effluent was withdrawn at a constant depth of 7 cm below the initial water level. The air flow rate in the aerobic phase was maintained at 8 L/min. The solid retention time (SRT) was maintained at 20 days by wasting 5% of the mixed liquor at the aerobic phase every day. The hydraulic retention time (HRT) was kept constant at 1.5 days, and the exchanged volume per cycle is 1.2 L, i.e. 1/3 of the liquid volume in the reactor. The dissolved oxygen (DO) and pH were measured but not controlled. There was a start-up period of three weeks before the commencement of regular water quality analysis (day 0), during which the reactor was fed with 4 times (×1/4) diluted wastewater (Table 1). During the first 42 days of the experiment, the SBR was fed with 4 times diluted effluent from the aforementioned precipitation reactor, then the dilution was reduced to 2 times (×1/2) in the subsequent 26 days. From 69 day onwards, the influent SBR was the precipitation reactor effluent without dilution. An anti-foam agent (Kebio RT500, Beijing, China), as suggested by (Tuantet et al. 2014), was added into the influent (0.025%, v/v) to prevent excessive foams in the aerobic phase.

### Characterization of activated sludge in SBR

During the experiment, granular sludge appeared in SBR. Particle size distribution analysis of granular sludge collected on day 130 was performed on a mastersizer (Mastersizer 2000, Malvern Instruments Ltd, Malvern, UK). According to the Mastersizer’s instruction, activated sludge samples were dispersed in water in the Hydro 2000s module until a laser obscuration of 10–15% was established prior to the measurement. Each sample was analyzed in triplicate.

Fluorescence in situ hybridization (FISH) technique was employed to characterize the nitrifying bacteria of the granular sludge. Granules were fixed in 4% (w/v) paraformaldehyde in phosphate-buffered saline (PBS; 10 mM sodium phosphate buffer, 130 mM sodium chloride; pH 7.2) at 4 °C for 3 h, washed three times with PBS, and embedded in Tissue-Tek OCT compound (Sakura Finetek, Torrance, CA) at 30 °C overnight. Sections measuring 10–20 μm in thickness were prepared using a cryostat (Reichert Jung Cryocut 1800, Leica, Bensheim, Germany). FISH was performed with a EUBmix (EUB338, EUB338-II, EUB338-III) primer specific for the members of the domain Bacteria, NSO1225 specific for all ammonia-oxidizing β-*proteobacteria*, and NIT3, Nstpa662, Ntcoc206 and Ntspn693 specific for *Nitrobacter*, *Nitrospira*, *Nitrococcus* and *Nitrospina*, respectively. The 16S rRNA-targeted oligonucleotide probes used in this study are listed in Additional file 1: Table S1. Hybridized samples were observed using a LSM510 confocal laser-scanning microscope (CLSM, Carl Zeiss, Oberkochen, Germany) equipped with an Ar ion laser (488 nm) and He–Ne laser (543 nm).

To reveal the microbial diversity of granular sludge, biomass samples for high-throughput sequencing analysis were harvested on day 133. DNA of biomass samples were extracted using the E.Z.N.A. soil DNA kits (Omega, USA) following the manufacturer’s instructions and then pooled together for further molecular analysis. The bacterial 16S rRNA gene was amplified with 338F and 806R primers (Xing et al. 2016). Amplicons were purified and sequenced on an Illumina Miseq platform at Majorbio Bio-pharm Technology Co. Ltd., Shanghai. After quality filtration, 27,510–34,693 effective reads were obtained for each sample. Illumina sequencing data were archived at NCBI Sequence Read Archive under accession number SRR2167764.

### Analytical methods

COD and TN were measured photometrically with vial tests (Hach Lange, Shanghai, China). NH_4_^+^–N, NO_2_^−^–N and NO_3_^−^–N, TP, mixed liquor suspended solids (MLSS), sludge volume index (SVI) were measured according to standard methods (APHA 2005). Because nitrite exerts a COD of 1.1 g-COD/g-NO_2_^−^–N, the COD values were corrected accordingly.

## Results

### Treatment performance

**Fig. 1:**
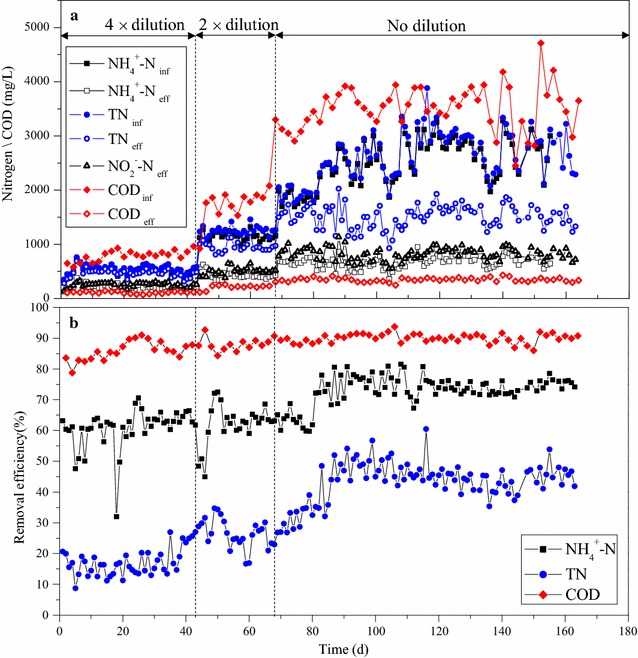
Concentrations (**a**) and removal efficiencies (**b**) of COD, ammonium nitrogen (NH_4_^+^–N), nitrite nitrogen (NO_2_^−^–N), and total nitrogen (TN) in the SBR

In the present study, the feed to SBR was the effluent from a precipitation reactor, in which undiluted source-separated urine and RO brine were mixed at a volumetric ration of 1:1. The TP concentrations in effluent of precipitation reactor varied in the range of 11.5–25.3 mg/L (Additional file 1: Figure S1). Precipitation reactor effluent also contained high concentration of organic carbon (COD of 2200–4800 mg/L) and the COD/N ratio varied from 1.05 to 1.85 (Fig. 1). To acclimate the biomass in the SBR reactor, the strength of influent was elevated from 4 times, then 2 times diluted urine, and finally to undiluted urine, thus the COD and nitrogen loads increased stepwise up to 3.23 kg COD/(m^3^ d) and 1.86 kg N/(m^3^ d), respectively. As shown in Fig. 1, during the first 42 days, i.e. the SBR fed with 4× diluted urine, the influent COD was kept around 700 mg/L, and the removal efficiencies attained 80–90%. Further increases and the fluctuations in influent COD hardly changed the removal efficiencies, and the effluent COD values of 200–400 mg/L were maintained throughout the experiment.

**Fig. 2:**
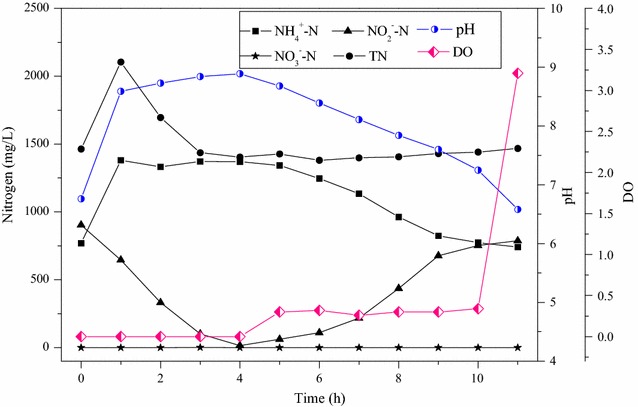
Profiles of nitrogen species, pH and dissolved oxygen (DO) in a typical cycle of the SBR

**Fig. 3:**
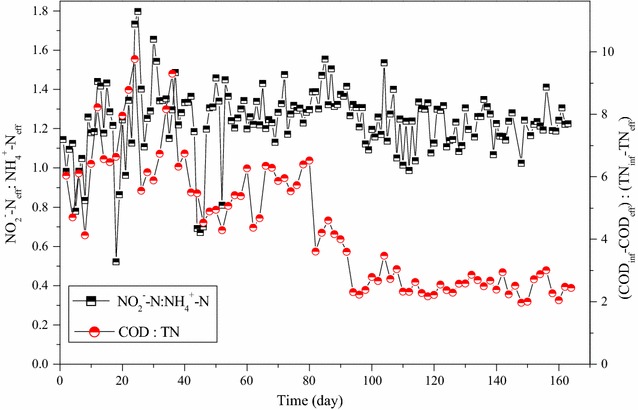
The NO_2_^−^–N:NH_4_^+^–N in the SBR effluent and the COD:TN removed in the reactor

In the stored urine, ammonium nitrogen constitutes more than 85% of the total nitrogen. 63 ± 8% of NH_4_^+^–N removal was achieved during the startup period of the SBR operation, while the removal efficiency was further improved to 75 ± 3% when the undiluted urine was fed into the reactor. The partial nitrification process led to the decrease of pH value from 9.0 ± 0.2 to 6.8 ± 0.3 (Fig. 2), and the extent of nitritation was limited by the alkalinity of the urine solution, which was reduced from 7 to 10 g-CaCO_3_/L down to 300–500 mg-CaCO_3_/L. Nitrite was the major oxidized species of nitrogen, while nitrate concentration was rarely detected and its concentration was less than 2 mg/L. The average concentrations of NH_4_^+^–N and NO_2_^−^–N in the effluent of SBR treating undiluted urine were 682 ± 121 and 815 ± 105 mg/L, respectively, resulting in a NO_2_^−^–N:NH_4_^+^–N of 1.24 ± 0.13 (Fig. 3).

The configuration of anoxic-aerobic conditions in SBR resulted in around 40% of nitrogen removal efficiencies under the steady state operations (Fig. 1). The reduction of TN occurred in the anoxic conditions, in which the nitrite produced in the previous cycle was denitrified with the influent organic matters as the carbon sources (Fig. 2). Although very low dissolved oxygen concentration (<0.3 mg/L) prevailed in the aerobic condition, no significant simultaneous nitrification and denitrification was observed. The organic carbon for nitrogen removal via nitrite, or so-called short-cut nitrification–denitrification, in the SBR was maintained at 2.57 mg-COD/mg-N removed (Fig. 3).

### Properties of biomass in SBR

**Fig. 4:**
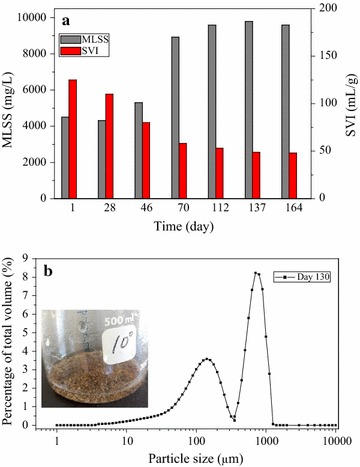
**a** The evolution of mixed liquor suspended solids (MLSS) and sludge volume index (SVI) for the biomass in the sequencing batch reactor. **b** The particle size distribution of the microbial aggregate sample taken on day 130. The image of granular sludge (*inset* **b**)

The biomass content measured by MLSS increased gradually from around 4000 to 10,000 mg/L, while the settleability of the sludge, in terms of SVI, improved from 120 to 48 mL/g (Fig. 4a). During the SBR operation, granular sludge was observed from the around 50 day and became the main microbial aggregates (Fig. 4b inset). The size distribution of the biomass in the reactor indicated there are two peaks on the volumetric percentage profile for these bio-aggregates, approximately at 100 and 1000 μm, respectively (Fig. 4b).

**Fig. 5:**
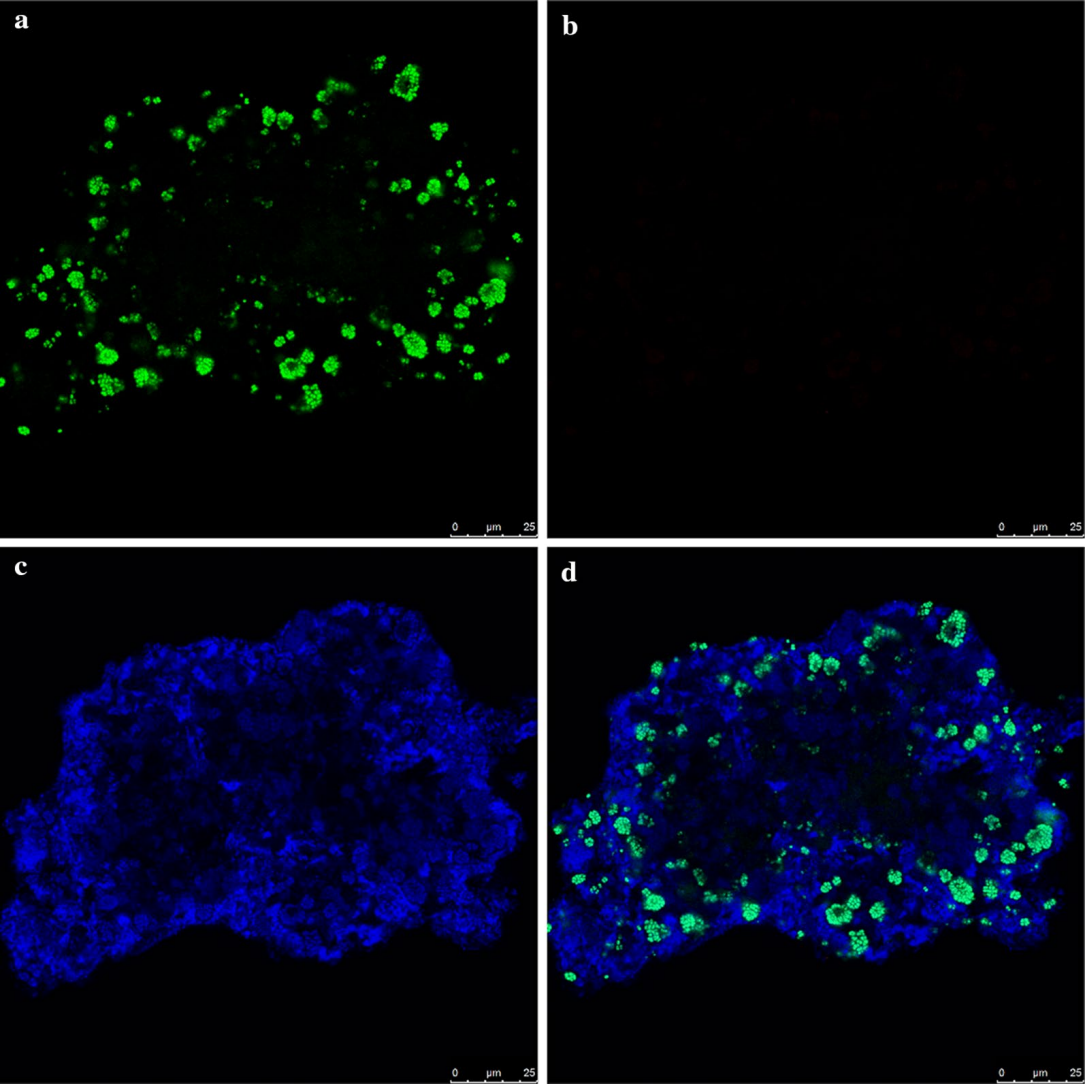
FISH micrographs (samples taken on day 154): **a** Nso1225 probe targeting ammonia-oxidizing bacteria was labeled in *green*, **b** NIT3, Nstpa662, Ntcoc206 and Ntspn693 specific for nitrite-oxidizing bacteria, **c** EUBmix probe targeting most bacteria as labeled in *blue*, **d** *three color* merged image, **a**–**c** represent the same zone in the cross section of a granule. *Bars* are 25 μm for **a**–**d**

FISH–CLSM was used to evaluate the enrichment of AOB and the presence of NOB in the granular sludge performing the partial nitritation–denitritation (Fig. 5). The total bacteria, targeted by probe mix EUB I–III, distributed homogeneously within the granular aggregates. Most AOB (*Nitrosomonas*-like) clusters, scattered in heterotrophic bacteria, were detected within a depth of approximately 25 μm in the periphery of the aggregate, while NOB were absent in the granular sludge. The abundance of AOB was 7.2% for the granular samples.

**Fig. 6:**
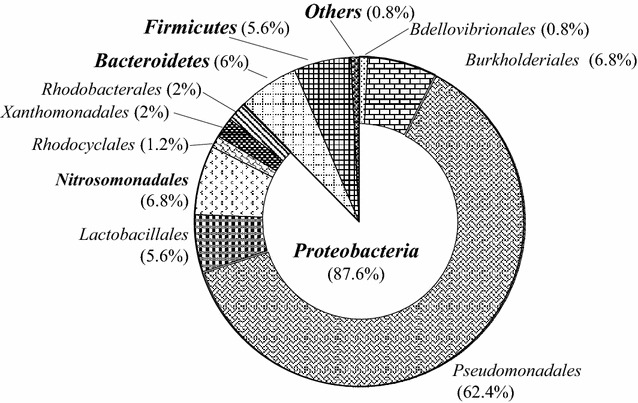
Microbial community composition of the activated sludge in SBR, according to a high throughput sequencing data. The inner pie chart shows the community composition at the phylum level, while the outer pie chart shows the community composition at the genus level for the phylum *Proteobacteria*. Relative abundance was calculated only considering those microorganisms in which the number of reads higher than 0.5% of the total reads

The 16S rRNA gene clone library analysis was carried out to determine the microbial community composition of the granules formed in the reactor. Among the bacterial populations identified based on the 16S rRNA sequence reads (n = 36,279), the majority belonged to *Proteobacteria* with relative abundance of 87.6% (Fig. 6). *Bacteroidetes* was the second phylum in order of abundance, with a value of 6.0%, followed by *Firmicutes* representing the 5.6% of the total reads. At the genus level, the reads related *Pseudomonas* accounted for 62.4% of the total reads, other reads relevant to heterotrophic bacteria, such as *Burkholderia*, *Lactobacteria*, *Rhodobacteria* spp. contributed to more than 15% of the total reads. The sequence reads related to *Nitrosomonas* AOB were identified and accounted for 6.8%. No clones related to NOB and anammox bacteria was observed.

## Discussion

Urine contains high contents of organic matters and nitrogen, but their concentrations are not high enough to economically recover these materials. Biological treatment technologies are generally regarded as the best approach to remove biodegradable organic and nitrogen compounds. Due to the low COD/TN ratios, neither conventional nitrification and denitrification nor the nitritation and denitritation appears effective to eliminate the nitrogen compounds in stored urine. PN-anammox could be an ideal option, but the presence of high concentration of organic matters is a challenge. Many previous studies reported that the biodegradable organic matters inhibit the anammox process in single stage PN-anammox in both activated sludge and biofilm reactors (Jenni et al. 2014; Liang et al. 2014; Tomar et al. 2015; Chen et al. 2016). The activity and abundance of anammox bacteria decrease under elevated COD/TN ratio conditions, since heterotrophic bacteria grow far faster than AMX and decreased the SRT of AMX in the reactors (Jenni et al. 2014; Liang et al. 2014). It is suggested to reduce the nitrogen loading rate for single-stage PN-anammox reactor for high COD/TN wastewaters. Therefore, two-stage PN-anammox, i.e. separated reactors for partial nitritation and anammox, respectively, would be preferred for waste-streams containing high concentrations of nitrogen and organics, for instance urine. It is reported that acetate accounts for about 50% of the total COD (Jenni et al. 2014). Since most organic compounds in the stored urine are easily biodegradable, they could be used as the carbon sources for denitritation. In the present work, most COD was used in the anoxic condition of the SBR to reduce nitrite to nitrogen gas (Fig. 3), reducing the overall nitrogen load and eliminating the inhibitive effect on the subsequent anammox process. Previous studies on biological nitrogen removal from source-separated urine have confirmed that ammonia volatilization and nitrous oxides emission account for less than 5% of the total nitrogen losses during the process (Udert et al. 2003; Burgmann et al. 2011; Macky et al. 2016).

Due to the hydrolysis of the urea present in the fresh urine during the storage, ammonium is released to be the major form of nitrogen in the stored urine, meanwhile leading to a rise of pH to around 9.0. In spite of high pH values, the alkalinity in stored urine is the limiting factor for the biological nitrification process, and only sustains about 50% ammonium oxidation by nitrifying bacteria (Udert et al. 2003), resulting in a decrease of pH to 6.5–7.2. Previous studies confirmed that the product of urine nitrification is either an ammonium-nitrate or ammonium-nitrite solution with an approximate 1:1 composition (Udert et al. 2003; Johansson and Hellstrom 1999). In the present work, we obtained an effluent of ammonium-nitrite solution, in similar to the SBR reactor operated by Udert et al. (2003), but the ratio of NO_2_^−^–N:NH_4_^+^–N was 1.24:1. Because the organic matters were used to reduce nitrite to N_2_ and to achieve around 40% total nitrogen removal, the buffer capacity produced in the denitritation process promoted the ammonium removal efficiency to 75 ± 3% as shown in Fig. 1. The composition of ammonium/nitrite (1:1.24) in SBR effluent is closed to the theoretical stoichiometry, i.e. 1:1.32, of the anammox process (Strous et al. 1998).

Granular sludge was formed and higher biomass was retained in the reactor, which significantly supports the growth of AOB and the high ammonium conversion rate of 1.39 kg-N/(m^3^ d) despite the high organic loading rate of 3.23 kg-COD/(m^3^ d). Pulse feeding, short settling time and strong shear force have been demonstrated as key factors in promoting the granulation of aerobic activated sludge (Beun et al. 1999; Martins et al. 2003; Qin et al. 2004). In this study, these factors played an important role in developing the discrete compact aggregates. Pulse feeding caused dramatic changes of substrate concentration and pH values in a cycle of SBR operation (Fig. 2), and enhanced substrate penetration into sludge aggregates and substrate storage at the beginning of each cycle. Moreover, heterotrophic bacteria underwent cyclic feast and famine conditions for substrates in SBR operation, which is regarded as a critical approach for granulation owing to the excessive production of extracellular polymeric substances (Tay et al. 2001). Short settling times exerted a hydraulic selection pressure on small flocs of activated sludge, and forced the slow-growing nitrifying communities acclimated in the granular aggregates. The high aeration flow rate (8 L/min) produced a superficial air upflow velocity of 3.68 cm/s and a shear rate of 1200 1/s. This high shear rate contributes to the formation of granular aggregates that resist deformation under physical stress.

FISH images indicate *Nitrosomonas* species were clustered into tight colonies and located in the outer regions of the aggregates, while the total bacteria exhibited a rather homogenous distribution throughout the granular aggregates, which implies a complete penetration of organic matters in the granular sludge (hundreds of μm). On the contrary, AOB were mainly present near the surface (<25 μm), and were surrounded by other bacteria. Song et al. measured the DO concentration profiles in granules performing PN, and showed that oxygen concentration decreased rapidly within 100 μm of the surface under the conditions of bulk DO concentrations of 2.5–7.0 mg/L (Song et al. 2013). Since the DO in our work was much lower (0.3 mg/L), the oxygen penetration region was far shallow.

NOB were not detected in the granular aggregates though the granules could retain slow-growing strains from washout. The absence of NOB in the microbial aggregates could partially be explained by the alternating inhibitions of free ammonia (FA, ~140 mg-NH_3_-N/L) and free nitrous acids (FNA, ~0.15 mg-HNO_2_-N/L) at the beginning and the end of the aerobic phases, respectively (Anthonisen et al. 1976). These values are much higher than the inhibition thresholds on NOB reported previously. Kim et al. pointed out 0.1–1.0 mg/L of FA will inhibit NOB, while AOB exhibit a much stronger tolerance to FA, withstanding concentrations up to 10–160 mg/L (Kim et al. 2005). Vadivelu et al. (2006) reported that NOB is thoroughly inhibited when concentration of FA and FNA reach 6 and 0.02 mg/L, respectively. Low DO concentration exerts a competitive inhibition by AOB over NOB due to the higher half-saturation coefficient of DO affinity for NOB (Picioreanu et al. 1997). NOB were more severely inhibited under low DO conditions than AOB, which partially account for the absence of NOB in granular activated sludge. Apart from nitrifying organisms, clones related with various potential denitrifiers, such as *Pseudomonas* spp. *Burkholderia* and *Lactobacillus*, were observed. Clones associated with *Pseudomonadales*, *Burkholderiales* and *Lactobacillales* bacteria accounted for more than 70% of the library.

In conclusion, 40% of total nitrogen removal could be obtained using the organic matters in stored urine as the electron donors in a SBR performing the partial nitritation and denitrification process. SBR produced an ammonium nitrite solution with a NO_2_^−^–N:NH_4_^+^–N of 1.24 ± 0.13, which is an ideal composition for anammox process. The biodegradable organic matters were effectively removed and their adverse impacts on anammox bacteria were eliminated. Granular sludge was formed in the SBR reactor, and the MLSS concentrations were up to 9.5 g/L. As a result, a high nitritation rate could be achieved under the volumetric loading rates of 3.23 kg-COD/(m^3^ d) and 1.86 kg N/(m^3^ d), respectively. *Nitrosonomas*-like AOB appeared in the outer regions of the granular aggregates, and NOB were not detected. Heterotrophic bacteria were homogeneously distributed in the granules. The microbial diversity analysis with the high throughput sequencing technique shows that *Proteobacteria* is the predominant phylum, in which *Pseudomonas* is the most abundant genus.

## Additional file

**Additional file 1.** Additional table and figure.

## Abbreviations

PN: partial nitritation
anammox: anaerobic ammonium oxidation
SBR: sequencing batch reactor
COD: chemical oxygen demand
P: phosphorus
N: nitrogen
AOB: ammonium-oxidizing bacteria
N_2_: di-nitrogen gas
AMX: anammox bacteria
NOB: nitrite-oxidizing bacteria
HET: heterotrophic bacteria
COD/N: chemical oxygen demand to nitrogen ratio
RO: reverse osmosis
VSS: volatile suspended solid
MLSS: mixed liquor suspended solids
SVI: sludge volume index
SRT: solid retention time
HRT: hydraulic retention time
DO: dissolved oxygen
FISH: fluorescence in situ hybridization
PBS: phosphate-buffered saline
CLSM: confocal laser-scanning microscope
FA: free ammonia
FNA: free nitrous acids

## References

[CR1] Anthonisen AC, Loehr RC, Prakasam TBS, Srinath EG (1976). Inhibition of nitrification by ammonia and nitrous acid. J Water Pollut Control Fed.

[CR2] APHA (2005). Standard methods for the examination of water and wastewater.

[CR3] Beun JJ, Hendeiks A, van Loosdrecht MCM, Morgenroth E, Wilderer PA, Heijnen JJ (1999). Aerobic granulation in a sequencing batch reactor. Water Res.

[CR4] Burgmann H, Jenni S, Vazquez F, Udert KM (2011). Regime shift and microbial dynamics in a sequencing batch reactor for nitrification and anammox treatment of urine. Appl Environ Microbiol.

[CR5] Chamchoi N, Nitisoravut S, Schmidt JE (2008). Inactivation of anammox communities under concurrent operation of anaerobic ammonium oxidation (ANAMMOX) and denitrification. Bioresour Technol.

[CR6] Chen C, Sun F, Zhang H, Wang J, Shen Y, Liang X (2016). Evaluation of COD effect on anammox process and microbial communities in the anaerobic baffled reactor (ABR). Bioresour Technol.

[CR7] Gomez J, Mendez R, Lema JM (2000). Kinetic study of addition of volatile organic compounds to a nitrifying sludge. Appl Biochem Biotechnol.

[CR8] Hanæus J, Hellström D, Johansson E (1997). A study of a urine separation system in an ecological village in Northern Sweden. Water Sci Technol.

[CR9] Jenni S, Vlaeminck SE, Morgenroth E, Udert KM (2014). Successful application of nitritation/anammox to wastewater with elevated organic carbon to ammonia ratios. Water Res.

[CR10] Johansson E, Hellstrom D (1999) Nitrification in combination with drying as a method for treatment and volume reduction of stored human urine. In: Johansson E (ed) Urine separating wastewater systems: design experience and nitrogen conservation. Licentiate Thesis. Lulea University of Technology, Lulea

[CR11] Kartal B, Kuenen JG, van Loosdrecht MCM (2010). Sewage treatment with anammox. Science.

[CR12] Kim DJ, Lee DL, Keller J (2005). Effect of temperature and free ammonia on nitrification and nitrite accumulation in landfill leachate and analysis of its nitrifying bacterial community by FISH. Bioresour Technol.

[CR13] Liang Y, Li D, Zhang X, Zeng H, Yang Z, Zhang J (2014). Microbial characteristics and nitrogen removal of simultaneous partial nitrification, anammox and denitrification (SNAD) process treating low C/N ratio sewage. Bioresour Technol.

[CR14] Macky HR, Morito GR, Hao T, Chen GH (2016). Pursuit of urine nitrifying granular sludge for decentralized nitrite production and sewer gas control. Chem Eng J.

[CR15] Martins AMP, Heijnen JJ, van Loosdrecht MCM (2003). Effect of feeding pattern and storage in the sludge settleability under aerobic conditions. Water Res.

[CR16] Maurer M, Pronk W, Larsen TA (2006). Treatment processes for source-separated urine. Water Res.

[CR17] Picioreanu C, Loosdrecht MCM, Heijnen JJ (1997). Modelling the effect of oxygen concentration on nitrite accumulation in a biofilm airlift suspension reactor. Water Sci Technol.

[CR18] Qin L, Tay JH, Liu Y (2004). Selection pressure as a driving force of aerobic granulation in sequencing batch reactors. Proc Biochem.

[CR19] Song Y, Ishii S, Rathnayake L, Ito T, Satoh T, Okabe S (2013). Development and characterization of the partial nitrification aerobic granules in a sequencing batch airlift reactor. Bioresour Technol.

[CR20] Strous M, Heijnen JJ, Kuenen JG, Jetten MSM (1998). The sequencing batch reactor as a powerful tool for the study of slowly growing anaerobic ammonium-oxidizing microorganisms. Appl Microbiol Biotechnol.

[CR21] Tay JH, Liu QS, Liu Y (2001). Microscopic observation of aerobic granulation in sequencing aerobic sludge blanket reactor. J Appl Microbiol.

[CR22] Tian X, Wang G, Guan D, Li J, Wang A, Li J, Yu Z, Chen Y, Zhang Z (2016). Reverse osmosis brine for phosphorus recovery from source separated urine. Chemosphere.

[CR23] Tomar S, Gupta SK, Mishra BK (2015). A novel strategy for simultaneous removal of nitrogen and organic matter using anaerobic granular sludge in anammox hybrid reactor. Bioresour Technol.

[CR24] Tuantet K, Temmink H, Zeeman G, Janssen M, Wijffels RH, Buisman CJN (2014). Nutrient removal and microalgal biomass production on urine in a short light-path photobioreactor. Water Res.

[CR25] Udert KM, Fux C, Munster M, Larsen TA, Siegrist H, Gujer W (2003). Nitrification and autotrophic denitrification of source-separated urine. Water Sci Technol.

[CR26] Udert KM, Larsen TA, Gujer W (2006). Fate of major compounds in source-separated urine. Water Sci Technol.

[CR27] Udert KM, Kind E, Teunissen M, Jenni S, Larsen TA (2008). Effect of heterotrophic growth on nitritation/anammox in a single sequencing batch reactor. Water Sci Technol.

[CR28] Vadivelu V, Keller J, Yuan ZG (2006). Effect of free ammonia and free nitrous acid concentration on the anabolic and catabolic processes of an enriched *Nitrosomonas* culture. Biotechnol Bioeng.

[CR29] Wilsenach J, Schuurbiers CH, van Loosdrecht MCM (2007). Phosphorus and potassium recovery from source separated urine through struvite precipitation. Water Res.

[CR30] Xing W, Li D, Li J, Hu Q, Deng S (2016). Nitrate removal and microbial analysis by combined micro-electrolysis and autotrophic denitrification. Bioresour Technol.

