# Supplementary material for: Partial nitritation of stored source-separated urine by granular activated sludge in a sequencing batch reactor
Source: AMB Express. 2017 Feb 28;7:50. doi: 10.1186/s13568-017-0354-9 (PMC5328902; doi:10.1186/s13568-017-0354-9)
Supplement: Supplementary file 1 — Additional file 1. Additional table and figure. [file 13568_2017_354_MOESM1_ESM.docx]

**Additional file**

**Journal name:** AMB Express

**Article title:** Partial nitritation of stored source-separated urine by granular activated sludge in a sequencing batch reactor

**Author names:** Liping Chen^1^, Xiaoxiao Yang^1^, Xiujun Tian^1^, Song Yao^1^, Jiuyi Li ^1,*^, Aimin Wang^1^, Qian Yao^2^, Dangcong Peng^2^

^1^ Department of Municipal and Environmental Engineering, Beijing Key Laboratory of Aqueous Typical Pollutants Control and Water Quality Safeguard, Beijing Jiaotong University, Beijing 100044, China

^2^ School of Environmental and Municipal Engineering, Xi’an University of Architecture and Technology, Xi’an 710048, China

*Corresponding author: J. Li [lijy@bjtu.edu.cn](mailto:lijy@bjtu.edu.cn)

Tel: +86-10-51684395, Fax: +86-10-51683764

Department of Municipal and Environmental Engineering,

School of Civil Engineering, Beijing Jiaotong University

Shangyuancun 3, 100044, Beijing, China.

Captions

1. Materials and methods-Phosphorus precipitation from stored urine using reverse osmosis (RO) brine as a precipitant

2. Fig. S1. Changes of total phosphorus (TP) concentration in the precipitation reactor and the sequencing batch reactor (SBR).

3. Table S1. FISH oligonucleotide probes used in the study.

**Materials and methods**

**Phosphorus precipitation from stored urine using reverse osmosis (RO) brine as a precipitant**

The RO brine used as a precipitant for phosphorus recovery in the present work was generated from the RO membrane unit in a thermal power plant located in the suburb of Beijing, China. The RO process desalinates the re-circulating cooling water, and the water recovery rate was maintained at 60 percent. The RO brine contains little suspended solids but high concentrations of dissolved ions, such as calcium and magnesium.

A laboratory-scale precipitation reactor was operated at room temperature (20 ± 2 °C) to recover the phosphorus in the stored urine. The P recovery reactor consisted of two cylinders in series to allow for precipitation reaction and solid - liquid separation, respectively. The effective volume of both cylinders was 1.3 L. The precipitation cylinder was operated in a continuously stirred tank reactor mode. An axial flow impeller was submerged half - way into the precipitation cylinder and stirred at 100 rpm. The stored urine and RO brine were added separately into the reactor at a volumetric ratio of 1:1. The hydraulic retention time (HRT) based on the volume of precipitation cylinder maintained constantly at 6 h. The effluent from settler for precipitates-urine separation was collected and used by the subsequent SBR to remove organic matters and nitrogen.

**Fig. S1.** Changes of total phosphorus (TP) concentration in the precipitation reactor and the sequencing batch reactor (SBR).

Table S1. FISH oligonucleotide probes used in the study.

| Probe name | Sequence (5’-3’) | Concentration ^a^ | Specificity |
| --- | --- | --- | --- |
| EUB_mix_ | EUB338: GCT GCC TCC CGT AGG AGT  EUB338-II: GCA GCC ACC CGT AGG TGT  EUB-III: GCT GCC ACC CGTAGG TGT | 35  40  35 | Eubacteria |
| NSO1225 | CGC CAT TGT ATT ACG TCT GA | *35* | Ammonia-oxidizing *β-proteobacteria* |
| Nsv443 | CCG TGA CCG TTT CGT TCC G | 30 | *Nitrosospira* |
| Nmv | TCC TCA GAG ACT ACG GGG | 35 | *Nitrosococcus* |
| Ntspa662 | GGA ATT CCG CGC TCC TGT | 35 | *Nitrospira* |
| NIT3 | CCT GGC TCC GCG CTC CTC T | 40 | *Nitrobacter* |
| NTCOC206 | CGG TGC GAG CTT GCA AGC | 10 | *Nitrococcus mobilis* |
| Ntspn693 | TTC CCA ATA TCA ACG CAT T | 20 | *Nitrospina gracilis* |

^a^ Concentrations presented as a volume percentage of formamide in hybridization buffer (%).
